# Prevalence of *Mycobacterium tuberculosis* and risk factors among internally and externally displaced populations in northwestern Ethiopia: The case of Dabat and Metema

**DOI:** 10.1016/j.ijregi.2025.100836

**Published:** 2025-12-30

**Authors:** Deresse Daka, Belay Tessema, Awelani Mutshembele, Amir Alelign, Wubet Birhan, Baye Gelaw

**Affiliations:** 1 Hawassa University College of Medicine and Health Sciences, Hawassa, Ethiopia; 2 University of Gondar, College of Medicine and Health Sciences, Department of Medical Microbiology, Gondar, Ethiopia; 3 University of Leipzig, Faculty of Medicine, Institute of Clinical Immunology, Leipzig, Germany; 4 South African Medical Research Council, South Africa Office of AIDS and TB, Pretoria, South Africa; 5 University of Gondar, College of Natural and Computational Science, Department of Biology, Gondar, Ethiopia

**Keywords:** *Mycobacterium tuberculosis*, Displaced populations, Refugees, Risk factors, Northwestern Ethiopia

## Abstract

**Objectives:**

Tuberculosis (TB) is a major global health crisis exacerbated by conflict and displacement. These factors disrupt health care and create overcrowded, unsanitary conditions that accelerate TB spread. This study investigated pulmonary TB epidemiology among refugees, internally displaced persons, and host communities in northwestern Ethiopia.

**Methods:**

A multicenter cross-sectional study was conducted from July 1 to September 30, 2024 at Dabat and Metema refugee sites in northwestern Ethiopia. In this study, 1350 sputum samples were tested using GeneXpert MTB/RIF, with culture performed on Löwenstein–Jensen medium. Data were analyzed in IBM SPSS Statistics, Version 27 using logistic regression, with model fitness assessed to ensure reliable findings.

**Results:**

Among 1350 study participants in northwestern Ethiopia, 56% were male, with a mean age of 35.29 years. The prevalence of Xpert MTB/RIF–confirmed TB was 102 cases (7.56%), of which 80 (78.4%) were culture-confirmed. Multivariable logistic regression identified several factors significantly associated with TB infection, such as cigarette smoking, recent TB contact, occupational exposure, diabetes, limited health care access, prolonged camp stay, alcohol consumption, biomass smoke exposure, weight loss, and shortness of breath.

**Conclusions:**

This study demonstrated a high prevalence of *Mycobacterium tuberculosis* among conflict-affected displaced populations. Diabetes mellitus, smoking, recent TB contact, alcohol use, biomass smoke exposure, prolonged camp residence, and poor health care access were identified as significant risk factors.

## Highlights

• High tuberculosis (TB) prevalence (7.6%) was found among conflict-affected displaced people.
• Prolonged camp residence and poor health care access elevate risk.
• The classic symptom hemoptysis was paradoxically associated with lower TB odds.
• Diabetes and smoking dramatically increase TB risk.

## Introduction

Tuberculosis (TB) is a leading global cause of death, concentrated in 30 high-burden countries, which account for 87% of cases. Only eight nations represent two-thirds of this total, led by India, and followed by Ethiopia, China, and Indonesia [1]. The disease disproportionately affects resource-limited settings, exacerbated by poverty and poor health care access, creating significant treatment barriers [2]. Ethiopia, as one of these high-burden countries, faces a high TB incidence and a severe HIV/TB co-infection epidemic, placing a substantial strain on its health care system [1].

During crises such as wars and forced displacement, the risk of TB can increase dramatically, up to 20-fold in some settings [3]. Armed conflicts severely disrupt health systems by damaging infrastructure, depleting medical supplies, and limiting access to care. Simultaneously, displacement leads to overcrowded and unsanitary living conditions that facilitate TB transmission [4]. Historical data support this trend: TB mortality rose during World War I because of reactivation of latent infections, while the conflict in Bosnia and Herzegovina saw a four-fold increase in new TB cases [5], and war in Congo-Brazzaville resulted in a two-fold increase in total TB cases [6]. These environments foster the spread and reactivation of both drug-sensitive and drug-resistant TB strains, posing significant challenges to disease control and public health response.

Displaced and refugee populations are significantly more vulnerable to TB, with the disease accounting for 30-50% of fatalities among Ethiopian and Chadian refugees in Sudan, and 26% among Ethiopian refugees in Somalia. TB severity is heightened among migrants, refugees, and internally displaced persons (IDPs) because of overcrowded shelters, poor sanitation, food insecurity, and limited access to health care services [7]. In addition to these structural challenges, refugees often face social stressors such as fear of harassment, while migrants may relocate in search of better living conditions or economic opportunities [8].

Northwestern Ethiopia, particularly the districts of Dabat and Metema, hosts a diverse range of displaced populations due to regional conflicts, climate-related stressors, and economic migration. These districts serve as critical humanitarian corridors along the Sudanese border, accommodating IDPs, refugees, host communities, and refugee-hosting communities. Evidence from previous studies indicates that the prevalence of TB among refugees in Ethiopian camps reaches 13.3%, markedly higher than the national average [8]. This study aimed to investigate the epidemiology of active pulmonary TB (PTB) across these four key population groups to better understand disease burden and inform targeted interventions.

## Methods and materials

### Study design

A cross-sectional study was conducted in the Dabat and Metema districts of northwestern Ethiopia between July and September 2024 and found that settlement camps provide refuge to displaced populations affected by conflict and instability. These regions host diverse displaced populations affected by conflict, climate stressors, and economic instability. Settlement camps in these districts serve as humanitarian corridors along the Sudanese border. Shimelaku Dabat Camp, established following the 2021 Tigray conflict, shelters 2220 IDPs from Tigray. The Alemwach refugee site accommodates 20,949 Eritrean refugees and 276 asylum seekers, integrated within a host community of 165,929 residents. Kumer Refugee Camp, located approximately 70 km from the Sudan border, primarily hosts 9383 Sudanese refugees, along with smaller groups from Eritrea and South Sudan [9].

### Study population and sampling

The study population comprised residents from the three camp sites and their adjacent host communities. A stratified random sampling technique was used. A pilot survey was conducted to refine the questionnaire, with feedback from subject-matter experts ensuring clarity, relevance, and reliability. The finalized questionnaire incorporated adjustments to improve comprehension and align with the study’s objectives. Trained health care professionals administered structured questionnaires through face-to-face interviews, ensuring standardized data collection and ethical consideration. Certified translators facilitated the interviews for participants unable to communicate in the primary study language by translating questionnaires into local languages. For individuals unable to self-report (e.g., children, cognitively impaired persons), responses were obtained through legally authorized guardians or proxies, adhering to ethical guidelines to ensure informed consent and data accuracy.

### Sample size determination and sampling technique

The sample size was determined at the cluster level (considering each displaced person [DP] site as a cluster) using a single-population proportion formula. On the basis of the reported prevalence of TB among vulnerable groups in the region, we assumed an expected prevalence of 13.4% (*P* = 0.134) [8]. Using an absolute precision of 5% and a 95% confidence interval (CI), the sample size was calculated using the single-population proportion formula:

$$n\;=\;\frac{{\left(Z_{\frac{\alpha }{2}}\right)}^{2}p\left(1-p\right)}{d^{2}},$$

where *n* is the sample size, Z_α/2_ is the significance level at α = 95%, *p* is the estimated prevalence from previous studies (the prevalence of TB among DPs in the Amhara Region was 13.36% [8]), and *d* is the margin of error (0.05). Accordingly, the required sample size was calculated as follows:

$$n\;=\;\frac{{\left(1.96\right)}^{2}0.134\left(1-0.134\right)}{0.05^{2}}=3.85\times 0.134\times 0.87/0.0025=179.53$$

Assuming an 80% response rate (due to poor documentation, refusal of participation, culture contamination, sample rejection, and other factors), the total sample size was *n* = 179 / 0.80 = 225. Hence, four DP sites were included: two hosting IDP and two hosting externally displaced refugees. These communities, comprising both internal and external migrants, were thus included in the study. Therefore, a total of 1350 participants (225 × six sites) were investigated for TB.

### Sample collection, storage, and transportation

Study participants who were suspected of having presumptive PTB were requested to submit spot or morning sputum specimens for molecular testing using the GeneXpert MTB/RIF assay (Cepheid). The sputum samples (3-5 ml) were collected from each study participant in a sterile screwcap 50-ml Falcon tube. The collected sputum samples were kept in a refrigerator until transported to the University of Gondar hospital TB laboratory. The sputum samples were transported in packaging consisting of a leak-proof primary receptacle (a 50-ml Falcon tube), leak-proof secondary packaging (the container that holds Falcon tubes), and a rigid outer package, as per World Health Organization category B packaging standards. The packages were constructed and closed to prevent any loss of contents that might be caused under normal transportation conditions by vibration or changes in temperature, humidity, or pressure. The specimens were delivered to the respective hospital laboratory within an hour of collection.

### Laboratory investigation and quality control

Direct Xpert Ultra MTB/RIF testing was performed on spot-sputum samples. The sample preparation involved mixing the spot sputum with sample diluent for 15 minutes, after which 2 ml of the specimen was transferred into the Xpert MTB/RIF cartridge and loaded into the GeneXpert instrument. The results were interpreted on the basis of the measured fluorescent signals and reported as detected, not detected, or invalid. Invalid results were managed by repeating the experiment. Xpert Ultra MTB/RIF–positive sediment was then neutralized, resuspended in 1-ml phosphate buffer solution, and inoculated onto Löwenstein–Jensen (LJ) medium (Becton Dickinson). The remaining sediment was stored in the refrigerator at –80°C. Inoculated LJ medium was incubated at 35-37°C for 6-8 weeks, with weekly examination for growth.

### Data processing and statistical analysis

Data were organized, cleaned, and analyzed using IBM SPSS Statistics, Version 27 (IBM Corp). Descriptive statistics were used to summarize the study variables. The prevalence of confirmed TB was expressed as a percentage. Factors associated with TB positivity among refugees were investigated using a logistic regression model. The adjusted odds ratio (AOR) and 95% CI were used as a measure of the strength of the association. Variables for the multivariable model were selected through a stepwise likelihood ratio approach, with non-significant predictors (*P* > 0.05) excluded from the final model. Descriptive statistics were used to summarize key study variables, and the prevalence of confirmed TB was reported as a percentage. To identify factors associated with TB positivity among refugees, a binary logistic regression model was applied. The strength of associations was measured using AORs with 95% CIs.

### Definition of terms

Refugee-residing communities (RCs) were defined as local populations living in proximity to refugee camps and sharing infrastructure and services.

## Results

### Socio-demographic characteristics of study participants

A total of 1350 study participants were included in the current study. Of these, 450 participants (33.3%) were recruited from Metema, and 900 participants (66.7%) were from Dabat. In the Metema cohort, 225 participants were refugees from Sudan, while 225 were permanent residents of Metema. Similarly, the Dabat cohort comprised 900 participants, with 225 individuals each drawn from the following groups: Dabat refugees, RCs, IDPs, and their hosting communities. All internally displaced participants were Ethiopian nationals originating from the Tigray Region. According to settlement status, half of the participants (675) were refugees, while the remaining half were members of host communities. The countries of origin of the study participants were Sudan (225, 16.7%), Eritrea (226, 16.7%), and Ethiopia (899, 66.6%). Most (756, 56.0%) were male, with a mean and median age of 35.29 (SD, 16.19) and 33 years, respectively. Approximately 32.5% had no formal education. The largest proportion of the study participants were farmers (31.5%). The median duration of residence in the refugee camp was 3 years (interquartile range = 2 years) (Table 1).

**Table 1:** Socio-demographic characteristics of study participants from northwestern Ethiopia, Amhara National Regional State, 2024.

Characteristics	Categories	Number	Percentage (%)
Sex	Male	756	56
	Female	594	44
Age, years	≤15	126	9.3
	15-24	228	16.9
	25-34	378	28.0
	35-44	260	19.3
	45-54	181	13.4
	≥55	177	13.1
Religion	Christian	1050	77.8
	Muslim	300	22.2
Marital status	Single	466	34.5
	Married	783	58.0
	Others	101	7.5
Educational level	Cannot read and write	439	32.5
	Can read and write	679	50.3
	Primary (grade 1-8)	117	8.7
	Grade 9-12 and above	115	8.5
Occupations (n = 803)	Formal employee	88	10.9
	Farmer	253	31.5
	Self-employee	73	22.4
	Student	117	14.6
	Unpaid domestic worker	163	20.3
	Informal labor	109	13.6

Most study participants showed classic TB indicators such as productive cough (1193, 88.4%), fever (1013, 75.0%), and night sweating (1034, 76.6%). Systemic signs such as loss of appetite (974, 72.1%) and weight loss (795, 58.9%) were also highly reported, indicating possible disease progression. Chest pain affected 915 (67.8%) patients, while hemoptysis (blood in sputum) was observed in 508 (37.6%). Shortness of breath was less common (572, 42.2%), suggesting variability in symptom presentation. Conversely, 1089 (80.0%) participants reported experiencing cough for >5 weeks. A history of TB infection was identified in 247 participants (18.3%). Alcohol consumption habits were reported by 779 individuals (57.7%). Additionally, among 1219 respondents who provided health care access information, 642 (52.7%) reported regular access to health care services (Table 2, Table 3).

**Table 2:** Clinical presentation of suspected pulmonary tuberculosis disease cases.

Symptoms	Yes, n (%)	No, n (%)
Cough	1350 (100%)	0 (0)
Productive cough	1193 (88.4%)	157 (11.6%)
Sputum with blood	508 (37.6%)	842 (62.4%)
Fever	1013 (75%)	337 (25%)
Night sweats	1034 (76.6%)	316 (23.4)
Loss of appetite	974 (72.1%)	376 (27.9%)
Loss of weight	795 (58.9%)	555 (41.1%)
Chest pain	915 (67.8%)	435 (32.2%)
Shortness of breath	572 (42.2%)	778 (57.6%)

**Table 3:** Health and lifestyle characteristics of internally and externally displaced and hosting populations in northwestern Amhara, 2024.

Characteristics	Response	Number	Percentage (%)
Duration of camp stay in years (n = 551)	<5	466	84.6
	≥5	85	15.4
Person per room (n = 225)	1	166	73.8
	>1	59	26.2
Coughing duration	<2 weeks	261	19.3
	≥2 weeks	1089	80.7
Household size	1-3	434	32.1
	4-5	589	43.6
	≥6	327	24.2
Smokers	Yes	63	4.7
	No	1287	95.3
History of TB	Yes	247	18.3
	No	1103	81.7
Have chronic disease	Yes	98	7.3
	No	1252	92.7
Have TB contact history in past 6 months	Yes	169	12.5
	No	940	69.6
	Not sure	241	17.9
TB patients in the family	Yes	93	6.9
	No	1257	93.1
Work in TB risky area	Yes	163	12.1
	No	1187	87.9
Knows HIV status	Yes	883	65.4
	No	467	34.6
Alcohol consumption habits	Yes	779	57.7
	No	571	42.3
Substance use (chat, cigarettes, etc.)	Yes	79	5.9
	No	1271	94.1
Been in jail or prison	Yes	35	2.6
	No	1315	97.4
Firewood or biomass smoke exposure	Yes	761	43.6
	No	589	52.7
Regular access to health care (n = 1219)	Yes	642	56.4
	No	577	47.3
Placed or refuged with known TB patients in the same cell	Yes	24	1.8
	No	1326	98.2

TB, tuberculosis.

### Prevalence of M. tuberculosis

The overall prevalence of Xpert MTB/RIF–confirmed TB in the study population was 7.6% (102/1350; 95% CI: 6.27-9.1%). Of the 102 Xpert-positive cases, 78.4% (80/102) were culture-positive, 18.6% (19/102) were culture-negative, and 2.9% (3/102) were contaminated. However, variation in TB prevalence was observed among the study sites: 5.2% (47/900) in Dabat and 12.2% (55/450) in Metema.

**Figure 1:**
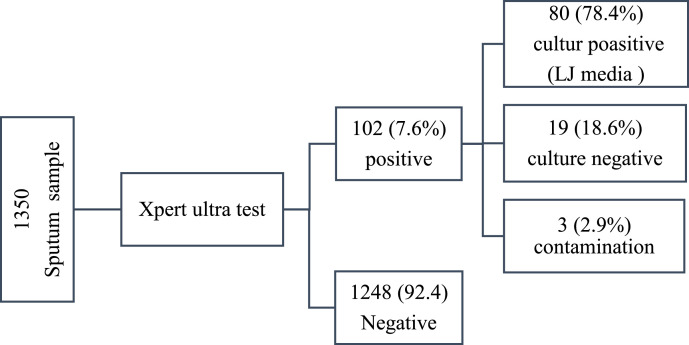
Flowchart of sputum samples and laboratory results in northwestern Ethiopia, 2024. LJ, Löwenstein–Jensen.

Neither country of origin nor study site of the refugees was significantly associated (*P* > 0.05) with bacteriologically confirmed TB cases. Prevalence varied substantially by sector and residency status: the highest proportion of *M. tuberculosis*–positive cases was observed among RCs in Metema at 15.1% (34/225), while refugees from other countries (e.g., Sudan) in Metema had a prevalence of 9.3%. In contrast, refugees living in Dabat had a significantly lower prevalence of 3.6%. TB prevalence among hosting communities (4.4%, 59/1350) was comparable to that among internally and externally displaced populations (3.2%, 43/1350), with no statistically significant difference observed (*P* > 0.05) (Figure 1).

### Factors associated with TB positivity

Logistic regression analysis revealed that while multiple symptoms (fever, chest pain, loss of appetite, night sweats, productive cough) showed significant associations with *M. tuberculosis* positivity in crude models, only loss of weight (AOR = 1.93, 95% CI: 1.12-3.31, *P* = 0.017) and shortness of breath (AOR = 1.69, 95% CI: 1.09-2.63, *P* = 0.018) remained statistically significant independent predictors after adjustment for confounding variables. Sputum containing blood demonstrated a significant inverse association with *M. tuberculosis* positivity in the adjusted model (AOR = 0.53, 95% CI: 0.33-0.85, *P* = 0.008) (Table 4).

**Table 4:** Crude and adjusted ORs of symptoms associated with TB status from bivariate and multivariable logistic regression analyses.

Symptoms		TB result		Crude OR (95% CI)	*P* value	Adjusted OR (95% CI)	*P* value
		Positive	Negative				
Productive cough	Yes	95	1098	1.85 (0.85, 4.07)	0.124	1.64 (0.72, 3.73)	>0.05
	No	7	150	1			
Blood-containing sputum	Yes	32	476	0.74 (0.48, 1.14)	0.176	0.533 (0.33, 0.85)	**0.008**
	No	70	772	1			
Fever	Yes	87	826	2.017 (1.15, 3.54)	0.014	1.66 (0.88, 3.12)	>0.05
	No	15	322	1			
Night sweats	Yes	85	949	1.58 (0.92, 2.69)	0.097	0.81 (0.42, 1.57)	>0.05
	No	17	299	1			
Loss of appetite	Yes	84	890	1.88 (1.11, 3.17)	0.018	1.42 (0.79, 2.56)	>0.05
	No	18	358	1			
Loss of weight	Yes	77	718	2.27 (1.43, 3.62)	<0.001	1.93 (1.12, 3.31)	**0.017**
	No	25	530	1			
Chest pain	Yes	84	831	2.34 (1.39, 3.95)	<0.001	1.23 (0.65, 2.33)	>0.05
	No	18	417	1			
Shortness of breath	Yes	59	513	1.97 (1.31, 2.96)	<0.001	1.69 (1.09, 2.63)	**0.018**
	No	43	735	1			

CI, confidence interval; OR, odds ratio; TB, tuberculosis.

Multivariable analysis identified numerous independent predictors of TB positivity (Table 4). Current smokers exhibited markedly elevated odds of TB (AOR = 15.92, 95% CI: 4.01-63.15, *P* < 0.001), as did individuals with recent TB contact history (AOR = 13.73, 95% CI: 6.10-30.89, *P* < 0.001) and occupational exposure in health care/high-risk settings (AOR = 11.23, 95% CI: 5.83-21.64, *P* < 0.001). Diabetes mellitus was significantly associated with TB (AOR = 7.27, 95% CI: 1.37-38.43, *P* = 0.02), while alcohol consumption doubled the odds (AOR = 2.15, 95% CI: 1.12-4.11, *P* = 0.021). Biomass smoke exposure was associated with significantly increased odds of TB (AOR = 2.23, 95% CI: 1.98-8.95, *P* < 0.001). However, asthma and substance use were not significantly associated after adjustment (*P* > 0.05), suggesting confounding in crude estimates (Table 5).

**Table 5:** Multivariable analysis of TB predisposing factors among internally and externally displaced people of northeastern Amhara National Regional State.

Symptoms		TB result		Crude OR (95% CI)	*P* value	Adjusted OR (95% CI)	*P* value
		Positive	Negative				
Presence of diabetes mellitus	Yes	19	11	25.74 (11.86, 55.88)	<0.001	7.27 (1.37, 38.43)	**0.02**
	No	83	1237	1			
Presence of asthma	Yes	12	27	6.03 (2.96, 12.3)	<0.001	2.76 (0.82, 9.29)	0.101
	No	90	1221	1			
Smoking status	Current	12	13	15.12 (6.67, 34.29)	<0.001	15.92 (4.011, 63.15)	**<0.001**
	Former	16	23	11.39 (5.78, 22.49)	<0.001	14.81 (5.47, 40.09)	**<0.001**
	Never	74	1212	1			
Contact history in last 6 months	Yes	72	142	8.66 (4.63, 16.21)	<0.001	13.73 (6.10, 30.89)	**<0.001**
	No	17	884	0.33 (0.16, 0.69)	0.003	0.30 (0.13, 0.708)	0.06
	Not sure	13	222	1			
Working experience at health facility or other TB risk area	Yes	50	104	10.58 (6.82, 16.37)	<0.001	11.23 (5.83, 21.64)	**<0.001**
	No	52	1144	1			
Alcohol consumption habit	Yes	74	738	1.83 (1.17, 2.86)	0.009	2.15 (1.12, 4.11)	**0.021**
	No	28	510	1			
Substance use habit	Yes	11	68	2.098 (1.098, 4.116)	0.031	2.14 (0.751, 6.07)	0.155
	No	91	1180	1			
Biomass smoke exposure	Yes	78	683	2.69 (2.23, 7.59)	<0.001	2.23 (1.98, 8.95)	**<0.001**
	No	24	565	1			

CI, confidence interval; OR, odds ratio; TB, tuberculosis.

This study indicated that longer duration of stay in the camp (≥5 years) and lack of access to a health facility were significant independent risk factors for TB infection. Refugees who had resided in camps for ≥5 years had 3.04 times higher odds of TB infection (95% CI: 1.38-6.69, *P* = 0.006) compared with those residing in the refugee camp <5 years, while those without health facility access had 4.13 times higher odds of infection (95% CI: 1.99-8.58, *P* = 0.001) compared with those with health access (Table 6).

**Table 6:** Association between selected risk factors and TB infection among refugees: bivariate and multivariable logistic regression analysis.

Variables		TB result		COR (95% CI)	*P* value	AOR (95% CI)	*P* value
		Positive	Negative				
Duration of time in the camp	<5	22	436	1			
	≥5	11	82	2.66 (1.24, 5.69)	0.012	3.04 (1.38, 6.69)	**0.006**
Access to health facility	Yes	27	615	1			
	No	75	502	3.40 (2.16, 5.37)	<0.001	4.13 (1.99, 8.58)	**0.001**
Refuged with chronically coughing persons in the same room	Yes	4	40	1.23 (0.43, 3.52)	0.696		
	No	98	1208	1			
Sharing eating and drinking materials	Yes	65	486	2.75 (1.81, 4.19)	<0.001	1.61 (0.73, 3.51)	>0.05
	No	37	762	1			

AOR, adjusted odds ratio; CI, confidence interval; COR, crude odds ratio; TB, tuberculosis.

## Discussion

This study assessed the prevalence of bacteriologically confirmed PTB and its associated factors among conflict-driven internally and externally displaced populations in Ethiopia’s Amhara Region. The overall prevalence was 7.6%, which is consistent with findings from North Wollo, Ethiopia (7.2%) [10], and populations experiencing homelessness in Medellín, Colombia (7.9%) [11]. However, it exceeds prevalence rates reported in northern Ethiopia (5.5%) [12] and marginalized immigrant groups in Italy (2.7%) [13]. In contrast, the observed prevalence was lower than previously reported estimates among refugees in Ethiopia, including 13.34% [8] and 28.4% [14]. This might be due to health care disruption in the camp leading to undiagnosed cases, displacement severity, post-conflict deterioration, differences in sensitivity and specificity of diagnostic methodologies, and overcrowding and poverty among vulnerable populations. Differences in study population characteristics and sampling techniques could also contribute to the observed variations in TB distribution; for example, this study focused on refugees and relied only on spot-sputum collection methods.

Individuals reporting hemoptysis had 47% lower odds of TB (AOR = 0.533, 95% CI: 0.33-0.85, *P* = 0.008), which contrasts with classical TB symptomatology, wherein hemoptysis is considered a hallmark symptom [15]. This discrepancy may be explained by care-seeking bias, as hemoptysis often triggers prompt medical attention, leading to early treatment initiation before bacteriological confirmation [16]. Additionally, in conflict-affected regions, hemoptysis may more frequently result from alternative conditions such as trauma, pneumonia, or parasitic infections such as paragonimiasis, rather than TB. Furthermore, the association may reflect disease stage dynamics, whereby hemoptysis typically occurs in advanced TB with cavitary lesions, while the most severe cases may succumb before diagnosis, thereby introducing survivor bias into the data.

In this conflict-driven population, weight loss was significantly associated with higher odds of TB (AOR = 1.93, 95% CI: 1.12-3.31, *P* = 0.017). This is consistent with a study conducted in Ethiopia [12]. Similarly, a Southern African study identified weight loss as a significant predictor of TB among household contacts [17]. This is due to undernutrition weakening the immune system, impairing the body’s ability to fight off infection, and making it harder to prevent the development of active TB from latent infection, thus increasing the risk of severe illness.

Patients reporting shortness of breath had significantly increased odds of having TB, with an AOR of 1.69 (95% CI: 1.09-2.63; *P* = 0.018) compared with those without this symptom. This finding aligns with an Italian study that also identified dyspnea as a significant predictor of TB [18]. Conversely, Rakoczy et al. [19] found shortness of breath to be a negative predictor for TB, as it was more strongly linked to non-TB diagnoses. This inconsistency is likely attributable to study population, clinical setting, or diagnostic differences.

Diabetes mellitus emerged as a significant independent predictor of TB infection in this study. The analysis revealed that diabetes mellitus significantly increased the odds of TB (AOR = 7.27, 95% CI: 1.37-38.43; *P* = 0.02). This concurs with global evidence identifying diabetes mellitus as a key risk factor for TB due to its impact on immune function [20]. Additionally, a study in northeastern Ethiopia noted that comorbidities such as diabetes mellitus exacerbate TB burden in conflict-affected areas [10]. This could be due to hyperglycemia impairing both innate and adaptive immunity, thus disrupting antigen-presenting cell function, reducing T-cell responsiveness, altering cytokine profiles, and increasing bacterial load, thereby facilitating progression from latent to active TB [21].

Current smokers had significantly elevated odds of TB (AOR = 15.92, 95% CI: 4.01-63.15; *P* < 0.001), as did former smokers (AOR = 14.81, 95% CI: 5.47-40.09; *P* < 0.001). This is consistent with studies conducted in Ethiopia [22] and Taiwan [23,24]. Furthermore, a 2020 systematic review and meta-analysis showed that tobacco smoking significantly increased the risk of drug-resistant TB, with pooled odds ratios reported for current and former smokers [20]. This could be due to smoking facilitating TB bacterial invasion and replication by damaging the respiratory epithelium, impairing ciliary function, causing mucus hypersecretion, and reducing mucociliary clearance [25].

Recent contact (within 6 months) with patients with TB dramatically increased TB infection probability (AOR = 13.73, 95% CI: 6.10-30.89; *P* < 0.001). Comparable studies have shown that individuals who come into contact with index TB cases have an increased risk of infection [12]. Furthermore, studies conducted in Vietnam [26], southwestern Iran [27], Malawi [28], and Ethiopia [10] demonstrated a strong link between TB infection and prior contact with individuals diagnosed with the disease. This outcome may be linked to the rapid progression of TB bacteria, as the body’s initial response can differ.

Working in health care or other high-risk TB settings was associated with significantly elevated odds of TB infection (AOR = 11.23, 95% CI: 5.83-21.64; *P* < 0.001). This finding is consistent with research conducted in Indonesia [29] and Ethiopia [30], which also highlighted a strong association between TB infection and prior contact with known cases among health care providers. A systematic review further confirmed that exposure in health care environments or other high-risk settings substantially increases the likelihood of TB infection [31]. This is due to health care workers and others in crowded occupational settings facing repeated exposure to aerosolized bacteria, with disrupted health services and damaged facilities creating conditions for higher TB transmission and further increasing infection risk.

This study found that alcohol users were twice as likely to be infected with TB compared with non-users, with an AOR of 2.15 (95% CI: 1.12-4.11; *P* = 0.021). This is consistent with a study conducted in Japan [32]. Furthermore, a major meta-analysis concluded that alcohol consumption is a significant risk factor for TB, with a pooled relative risk of 1.35 [33]. This might be due to the fact that alcohol use compromises the immune system, heightening susceptibility to TB and accelerating disease progression. It disrupts the function of key immune cells such as macrophages and interferes with cytokine signaling, reducing the body’s ability to combat *M. tuberculosis*. Additionally, alcohol-related social behaviors often increase exposure to TB, contribute to poor adherence to TB treatment, and lead to diagnostic delays that exacerbate transmission in vulnerable populations.

In this study, individuals exposed to biomass smoke had more than twice the odds of TB infection compared with those not exposed (AOR = 2.23, 95% CI: 1.98-8.95; *P* < 0.001). Multiple studies across Asia and Africa, including India [34], Pakistan [35], and Mongolia [36], have consistently reported a significant association between household biomass fuel use and two- to three-fold increased odds of PTB. This may be explained by exposure to biomass smoke, which contains pollutants such as particulate matter, carbon monoxide, and formaldehyde. These pollutants can weaken the body’s immune system, impair macrophage function and mucociliary clearance, and thereby increase pulmonary susceptibility to bacterial infections and reduce airway bacterial clearance.

Individuals residing in the refugee camp for 5 years or more had a significantly higher risk of TB infection (AOR = 3.04, 95% CI: 1.38-6.69; *P* = 0.006). Similarly, earlier findings showed that a longer duration of residence among refugee and migrant populations is associated with a heightened risk of TB infection [37] due to vulnerabilities in camp settings. This outcome may be linked to prolonged residence in refugee camps, which increases TB susceptibility by extending exposure to key risk factors, including overcrowding, cumulative contact with active cases, poor ventilation, malnutrition, stress, and limited health care access [12,38].

In this study, limited access to health facilities was identified as the most significant risk factor for TB, with individuals lacking such access having over four times greater odds of testing positive (AOR = 4.13, 95% CI: 1.99-8.58; *P* = 0.001). This finding is supported by multiple studies indicating that high TB prevalence is linked to gaps in health care services [8,39]. This association may stem from geographical variation, financial barriers, socio-economic deprivation, and a weak local health system, which likely cause significant delays in diagnosis and treatment and impede preventive care.

### Limitations

This study has limitations, including its cross-sectional design, which cannot definitively determine causality between risk factors and TB infection; susceptibility to recall and social desirability bias; potential residual confounding from unmeasured variables; limited generalizability to stable populations or displaced settings; and wide CIs for strong associations, often due to a small sub-group sizes. In addition, the absence of comprehensive data on common co-infections such as HIV and intestinal parasites limits the ability to assess their potential modifying effects on TB risk.

## Conclusion

This study identified a high prevalence of PTB among conflict-displaced populations in northwestern Ethiopia. PTB infection was driven by a confluence of individual health conditions, most notably diabetes mellitus and a history of smoking. Profound structural determinants included prolonged camp residence, limited access to health facilities, occupational exposure, and household air pollution from biomass smoke. These findings emphasize the critical need for integrated public health interventions that combine enhanced clinical screening for at-risk individuals with broader strategies to improve living conditions, ensure health care access, and address the socio-environmental drivers of TB transmission in such humanitarian settings.

## Declaration of competing interest

The authors have no competing interests to declare.
